# DCAF7 regulates cell proliferation through IRS1-FOXO1 signaling

**DOI:** 10.1016/j.isci.2022.105188

**Published:** 2022-09-24

**Authors:** Scott Frendo-Cumbo, Taoyingnan Li, Dustin A. Ammendolia, Etienne Coyaud, Estelle M.N. Laurent, Yuan Liu, Philip J. Bilan, Gordon Polevoy, Brian Raught, Julie A. Brill, Amira Klip, John H. Brumell

**Affiliations:** 1 Cell Biology Program, Hospital for Sick Children, Toronto, ON M5G 0A4, Canada; 2 Department of Physiology, University of Toronto, Toronto, ON M5G 1L7, Canada; 3 Department of Molecular Genetics, University of Toronto, Toronto, ON M5G 1L7, Canada; 4 Princess Margaret Cancer Centre, University Health Network, Toronto, ON M5G 2C1, Canada; 5 Department of Medical Biophysics, University of Toronto, Toronto, ON M5G 1L7, Canada; 6 Institute of Medical Science, University of Toronto, Toronto, ON M5S 1A8, Canada; 7 Department of Biochemistry, University of Toronto, Toronto, ON M5G 1L7, Canada; 8 SickKids IBD Centre, Hospital for Sick Children, Toronto, ON M5G 1X8, Canada

**Keywords:** Genetics, Cell biology

## Abstract

Cell proliferation is dependent on growth factors insulin and IGF1. We sought to identify interactors of IRS1, the most proximal mediator of insulin/IGF1 signaling, that regulate cell proliferation. Using proximity-dependent biotin identification (BioID), we detected 40 proteins displaying proximal interactions with IRS1, including DCAF7 and its interacting partners DYRK1A and DYRK1B. In HepG2 cells, DCAF7 knockdown attenuated cell proliferation by inducing cell cycle arrest at G2. DCAF7 expression was required for insulin-stimulated AKT phosphorylation, and its absence promoted nuclear localization of the transcription factor FOXO1. DCAF7 knockdown induced expression of FOXO1-target genes implicated in G2 cell cycle inhibition, correlating with G2 cell cycle arrest. In *Drosophila melanogaster*, wing-specific knockdown of DCAF7/*wap* caused smaller wing size and lower wing cell number; the latter recovered upon double knockdown of *wap* and *dfoxo*. We propose that DCAF7 regulates cell proliferation and cell cycle via IRS1-FOXO1 signaling, of relevance to whole organism growth.

## Highlights

• DCAF7 is a novel interactor of IRS1 in mammalian cells
• DCAF7 regulates IRS1-FOXO1 signaling and cell proliferation in mammalian cells
• *Drosophila* DCAF7 homolog *wap* regulates wing size via *dfoxo*

## Graphical abstract

**Figure:**
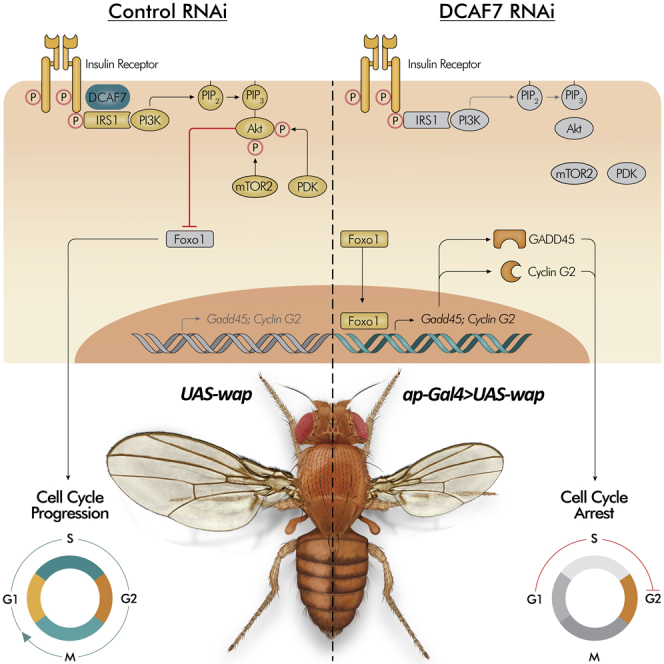

## Introduction

Cell proliferation is a key determinant of whole-organism growth, typified by progression through the cell cycle. In turn, movement through the cell cycle phases (G1, S, G2, and M) is defined by increased protein and DNA content required for cell division. G1 and G2 phases possess checkpoints at which the cell cycle can be halted if intracellular or environmental conditions are unsuitable for growth. This control is mediated by cell cycle inhibitors that are largely regulated at the transcriptional level. Progression through the cell cycle is further regulated by growth factors, notably insulin and insulin-like growth factor 1 (IGF1).

Insulin signaling has long been known to promote cell proliferation (Hill and Milner, 1985; Straus, 1981). Insulin and IGF1 initiate signaling upon cognate receptor binding, inducing receptor autophosphorylation that generates binding sites for insulin receptor substrate 1 (IRS1) (Copps and White, 2012; Taniguchi et al., 2006). In turn, IRS1 acts as a scaffold for phosphoinositide 3-kinase (PI3K), leading to AKT recruitment and activation by phosphorylation at S473 and T308 (Hemmings and Restuccia, 2012; Xu et al., 2012). One important mechanism involved in insulin/IGF1 regulation of cell proliferation involves AKT-mediated inhibition and nuclear exclusion of the transcription factor forkhead box O1 (FOXO1) (Accili and Arden, 2004; Kops et al., 2002; Medema et al., 2000; Xie et al., 2012). Active FOXO1 promotes gene expression of cell cycle inhibitors (CDKN1B, GADD45A, RBL2, and CCNG2), and thus growth factor-induced inhibition of FOXO1 promotes progression through the cell cycle (Carter and Brunet, 2007; Daitoku et al., 2011; Greer and Brunet, 2005).

Studies in *Drosophila melanogaster* provide a striking illustration of the importance of insulin signaling through the IRS1/FOXO1 axis in cell proliferation and development. Imaginal disc cells responsible for wing development in larvae exhibit cell cycle progression through its classical phases (Neufeld et al., 1998). Proliferation of these cells is dependent on *Drosophila* insulin-like peptides and mimicked by mammalian insulin (Brogiolo et al., 2001; Cullen and Milner, 1991; Edgar and Lehner, 1996). Moreover, loss of the *Drosophila* insulin receptor (*dInr*) or of *chico*, the IRS ortholog, leads to smaller body size due to a reduction in cell number (Chen et al., 1996; Oldham et al., 2000). Phenocopying the growth retardation observed upon *chico* depletion, overexpression of *dfoxo* diminishes growth in flies (Kramer et al., 2003). Connecting the pathway, *dfoxo* knockout partially restores growth in *chico* knockout flies by recuperating cell proliferation (Jünger et al., 2003).

In an attempt to map elements of this developmental pathway, we used proximity-dependent biotin identification (BioID) to screen the IRS1 interactome for protein regulators of cell cycle. We identified DDB1- and CUL4-associated factor 7 (DCAF7, also known as WDR68 or Han11), as an IRS1 interactor. DCAF7 is a scaffold protein previously recognized as an essential gene for cell survival and implicated in *Drosophila* development (Alvarado et al., 2016; Hart et al., 2015; Miyata and Nishida, 2011; Morriss et al., 2013; Nissen et al., 2006; Ritterhoff et al., 2010; Yang et al., 2016; Yousefelahiyeh et al., 2018). However, whether DCAF7 is required for cell proliferation and/or insulin signaling was unknown. Here, our data suggest that DCAF7 acts as a scaffold for IRS1-PI3K that is required for insulin signaling to AKT and FOXO1 inhibition. Furthermore, we reveal a conserved function for DCAF7 in regulating the cell cycle via FOXO1, fulfilling a role as an integral mediator of cell proliferation and whole-organism growth.

## Results

### Identification of DCAF7 as a proximity interactor of IRS1

**Figure 1:**
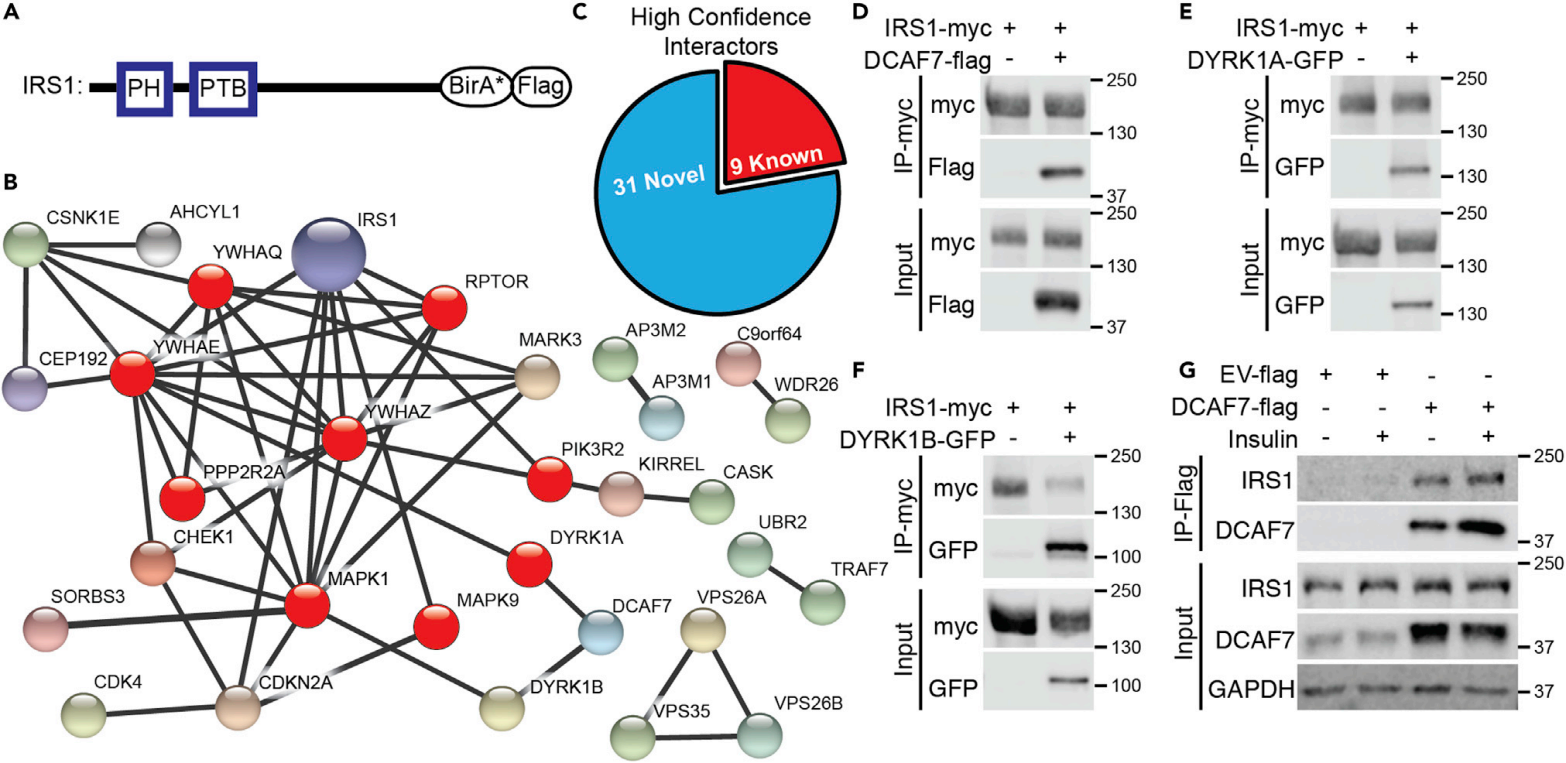
BioID identification of IRS1 proximity interactors (A) IRS1 domain structure and BirA∗-Flag C-terminus tag. (B) IRS1 BioID high-confidence proximity interactors. Connecting lines depict known functional and physical associations (STRING database). Known IRS1 interactors are depicted in red. (C) Classification of known and newly identified high-confidence proximity interactors of IRS1 from our BioID dataset. High-confidence proximity interactors were determined using SAINT analysis comparing IRS1-BirA∗ identified interactors to that of BirA∗ alone. (D–F) Coimmunoprecipitation of IRS1myc in HEK293T cells with DCAF7-Flag, DYRK1A-GFP, and DYRK1B-GFP. (G) Coimmunoprecipitation of DCAF7-Flag with endogenous IRS1, in the presence or absence of insulin, in HepG2 cells.

To gain insight into the regulation of cell proliferation, we characterized IRS1 protein proximity interactions using BioID. To this end, we used an abortive mutant of the *Escherichia coli*-derived biotin ligase BirA (R118G, termed BirA∗) (Choi-Rhee et al., 2008) that results in irreversible tagging of proximal proteins under physiological conditions. The IRS1 gene was cloned with a C-terminal BirA∗-Flag dual-tagged vector under control of a tetracycline-inducible promoter and stably expressed in human embryonic kidney (HEK) 293 cells (Figure 1A).

Compared to control data from Flag-BirA∗ BioID alone performed under similar conditions, proximity interactions emerged with 40 unique human proteins for IRS1-BirA∗-Flag ((1% FDR), Data S1). The resulting protein proximity interaction network includes high-confidence hits that can be clustered according to known physical and functional associations from the STRING database (www.string-db.org) (Figure 1B). The IRS1-BirA∗-Flag dataset included 9 proteins previously shown to interact with and regulate IRS1 (Figure 1C) (Craparo et al., 1997; De Fea and Roth, 1997; Gual et al., 2005; Hartley and Cooper, 2002; Liu et al., 2018; Mandavia and Sowers, 2012; Ogihara et al., 1997; Tuncman et al., 2006; Xiang et al., 2002), validating the use of BioID to identify binding partners of IRS1.

The IRS1-BirA∗-Flag dataset included 31 proximity interactions with proteins not previously known to bind IRS1. Of particular interest was the scaffold protein DCAF7, along with its binding partners dual-specificity tyrosine phosphorylation-regulated kinase 1A (DYRK1A) and DYRK1B (Miyata and Nishida, 2011; Yousefelahiyeh et al., 2018). Using IRS1-myc coimmunoprecipitation, we confirmed that IRS1 interacts with DCAF7-Flag, DYRK1A-GFP, and DYRK1B-GFP in HEK293T cells (Figure 1D–1F). The DCAF7-IRS1 interaction was further supported by coimmunoprecipitation of DCAF7-Flag in HepG2 cells and this interaction was unaffected by insulin stimulation (Figure 1G), suggesting that the interaction between DCAF7 and IRS1 is not regulated by insulin action. Moreover, coimmunoprecipitation of DCAF7-Flag supports that both DYRK1A-GFP and DYRK1B-GFP interact with DCAF7 in HepG2 cells. Conversely, siRNA-mediated knockdown of DCAF7 reduced DYRK1A levels (Figures S1A and S1B). HepG2 cells do not express detectable DYRK1B protein; however, DYRK1B protein abundance was also reduced in C2C12 muscle cells upon loss of DCAF7 (Yousefelahiyeh et al., 2018). Interestingly, the interaction of DYRK1A-GFP with IRS1-myc was diminished by loss of DCAF7, without changes in protein levels upon overexpression, suggesting that DCAF7 is required for optimal DYRK1A binding to IRS1 (Figure S1C).

DCAF7 was previously identified in a large-scale CRISPR knockout screen as an essential gene required for cell viability (Hart et al., 2015; Peng et al., 2016) and studies in various model systems, including *Drosophila*, found that loss or mutation of DCAF7 is associated with growth retardation (Alvarado et al., 2016; Morriss et al., 2013; Nissen et al., 2006; Yang et al., 2016). However, the contextual mechanism through which DCAF7 impacts growth remains unclear. We hypothesized that DCAF7 may regulate cell proliferation and that this influence is exerted at the level of the cell cycle.

### DCAF7 is essential for cell proliferation and G2 cell cycle progression

**Figure 2:**
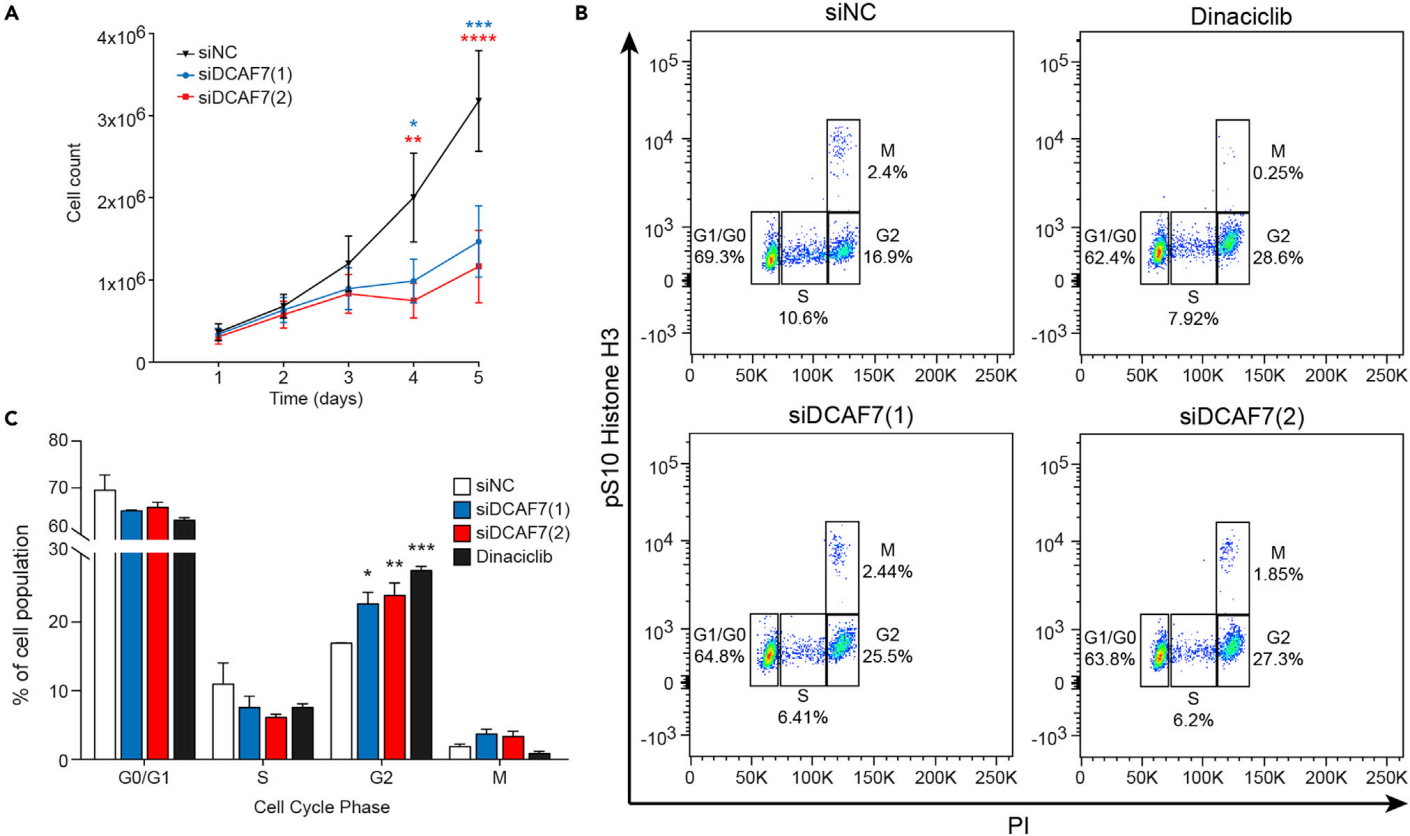
DCAF7 knockdown attenuates cell proliferation and promotes G2 cell cycle arrest (A) HepG2 cell number 1–5 days post siRNA transfection of NC or two DCAF7 siRNA (n = 5/group; mean ± SEM). Cells were grown in the presence of serum. p value was calculated using two-way ANOVA with Fisher’s LSD post-hoc. See also Figure S2. (B) Representative plots of cell cycle analysis following siRNA transfection. Transfected cells were fixed and stained with pS10 Histone H3-488 and PI prior to flow cytometry for cell cycle analysis. Cells treated with Dinaciclib (25 nM for 24 h), a pharmacological inhibitor of cell cycle at G2, were used as a positive control. (C) Quantification of the percent of cell population in each phase of the cell cycle (n = 3/group; mean ± SEM). p value was calculated using one-way ANOVA with Fisher’s LSD post-hoc. ∗p < 0.05, ∗∗p < 0.01, ∗∗∗p < 0.001, ∗∗∗∗p < 0.0001.

To explore the possible role of DCAF7 in cell proliferation, we performed DCAF7 knockdown and investigated changes in cell number and cell cycle. DCAF7 protein abundance was efficiently reduced in HepG2 cells up to 5 days post transfection with two DCAF7 siRNAs (Figure S2). Cell number was significantly lower on days 4 and 5 post transfection with DCAF7 siRNA (Figure 2A). To assess whether diminished cell proliferation was a result of cell cycle inhibition, we performed flow cytometry for S10-phosphorylated histone H3, a marker of mitosis, and propidium iodide (PI) to assess cellular DNA content. Knockdown of DCAF7 induced cell cycle arrest at G2, to an extent similar to that obtained with Dinaciclib (Figure 2B and 2C), a cyclin-dependent kinase inhibitor that provokes cell cycle arrest at G2 (Jane et al., 2016; Lin et al., 2017; Rajput et al., 2016). These findings suggest that DCAF7 knockdown blunts cell proliferation via G2 cell cycle inhibition.

### DCAF7 knockdown attenuates AKT phosphorylation and promotes expression of FOXO1-regulated genes involved in G2 cell cycle inhibition

**Figure 3:**
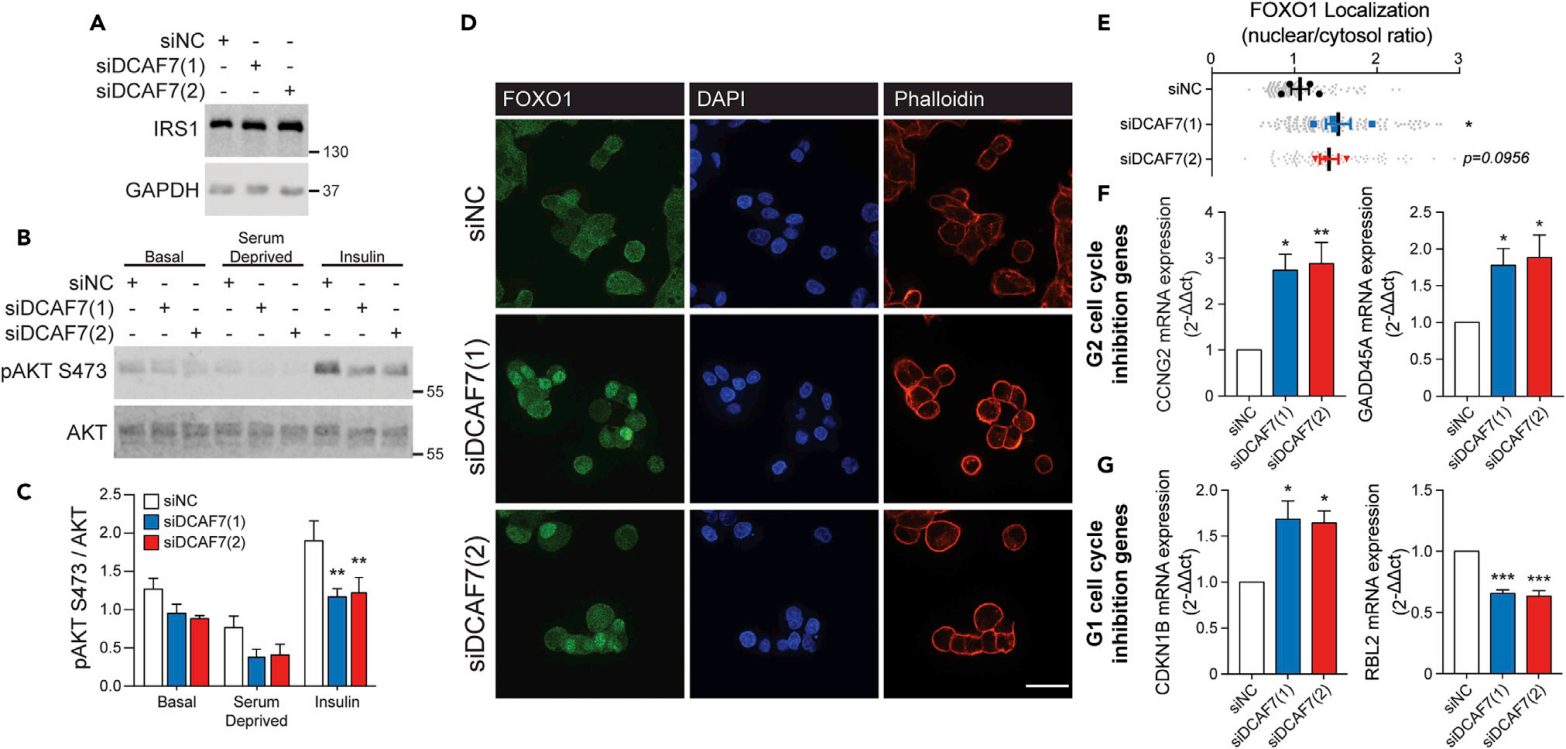
DCAF7 knockdown attenuates insulin-stimulated AKT phosphorylation and promotes FOXO1 nuclear localization and expression of FOXO1-regulated genes (A) HepG2 cells were transfected with non-coding (NC) or two independent DCAF7 siRNA 48 h prior to experimentation. IRS1 protein abundance was examined by Western blotting. See also Figure S2. (B and C) Cells were maintained in regular media (basal) or serum deprived for 3 h prior to treatment with insulin, and phosphorylation of AKT on S473 was examined by Western blotting (n = 3/group; mean ± SEM). p value was calculated using a two-way ANOVA with Tukey’s post-hoc. See also Figure S3. (D) FOXO1 localization in siRNA-transfected HepG2 cells (scale: 30 μm). Cells were serum deprived for 3 h prior to fixation. (E) Nuclear/cytosolic ratio of FOXO1 was analyzed using ImageJ software (n = 3–4/group; mean ± SEM). p value was calculated using one-way ANOVA with Fisher’s LSD post-hoc. (F and G) Expression of FOXO1-regulated cell cycle inhibition genes was examined by qPCR (n = 3–4/group; mean ± SEM). p value was calculated using one-way ANOVA with Fisher’s LSD post-hoc. ∗p < 0.05, ∗∗p < 0.01, ∗∗∗p < 0.001.

Based on our identification of DCAF7 as an IRS1 interactor and its importance in cell proliferation, we next examined whether DCAF7 affects insulin signaling mediators essential for cell cycle progression. For these experiments, HepG2 cells were depleted of serum for 3 h prior to stimulation with insulin to remove tonic activation of IRS1 by serum. While DCAF7 knockdown did not affect IRS1 protein abundance, loss of DCAF7 significantly diminished insulin-stimulated AKT S473 and T308 phosphorylation (Figure 3A–3C, S3A, and S3B). However, the phosphorylated levels of ERK, which are independent of the PI3K-AKT pathway, were not reduced by DCAF7 knockdown (Figure S3C). This suggests specificity of DCAF7 in IRS1-AKT signaling downstream of the insulin receptor.

Under control conditions, AKT activation inhibits FOXO1, promoting its nuclear exclusion and reduced transcriptional activity (Biggs et al., 1999; Brunet et al., 1999; Guo et al., 1999; Tang et al., 1999). Upon knockdown of DCAF7, FOXO1 exhibited enhanced nuclear localization (Figures 3D and 3E), that led to a significant increase in expression of FOXO1-target genes involved in G2 cell cycle arrest, namely growth arrest and DNA damage-inducible alpha (GADD45A) and cyclin G2 (CCNG2) (Figure 3F). In contrast, genes involved in G1 cell cycle arrest were variably affected, with CDKN1B (p27) increasing following DCAF7 knockdown and RBL2 (p130) decreasing (Figure 3G). In contrast to the observed changes in FOXO1, insulin-stimulated P70S6K phosphorylation, which occurs downstream of mTORC1 and is implicated in regulating cell size and cell cycle (Fenton and Gout, 2011), was not reduced by DCAF7 knockdown (Figure S3D). Together, these findings suggest upregulation of FOXO1 activity following knockdown of DCAF7 may be responsible for cell cycle inhibition at the level of G2.

### dFOXO mediates growth defects observed upon *wap* depletion by RNAi in *Drosophila*

**Figure 4:**
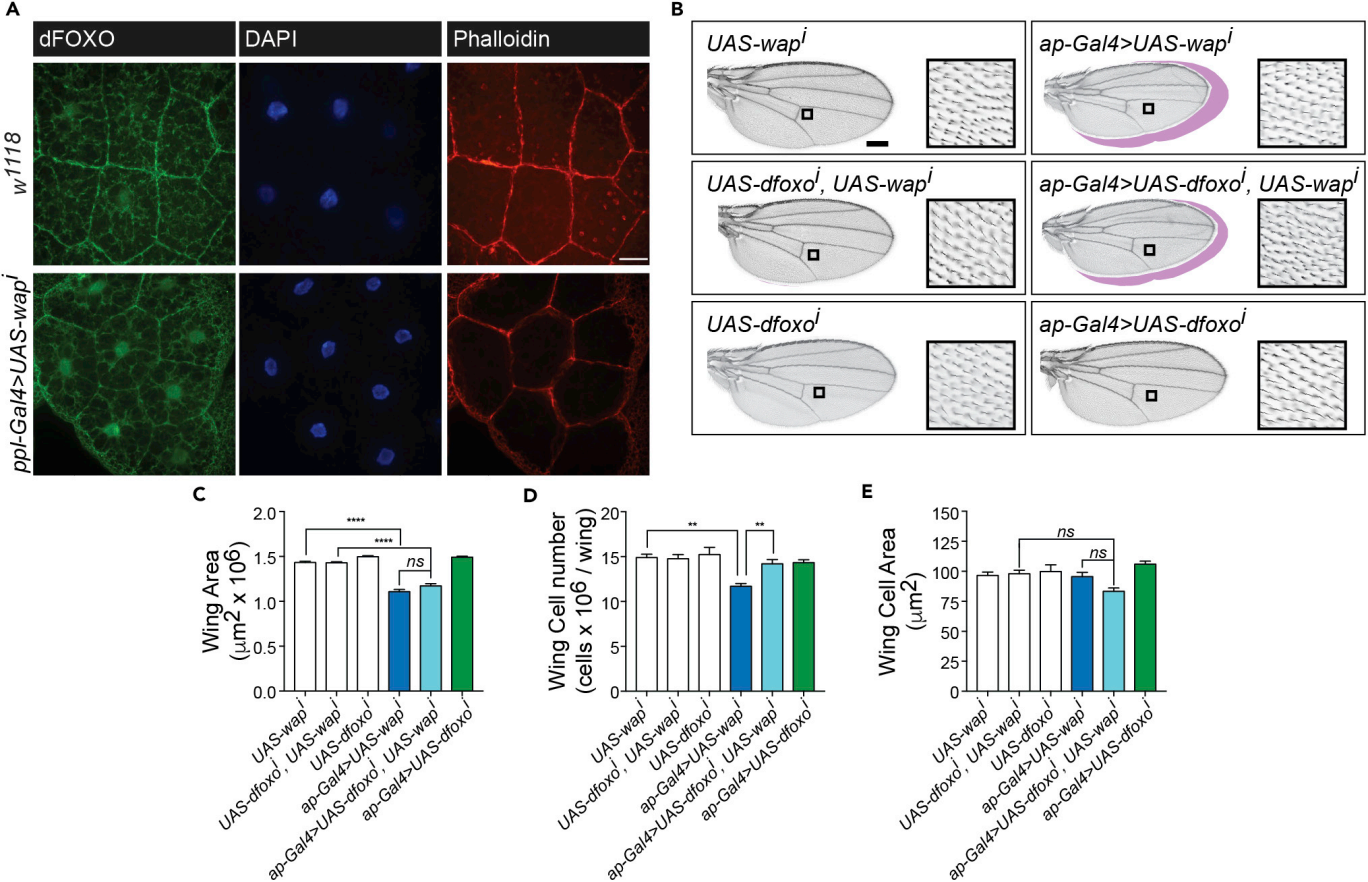
*wap* knockdown induces dFOXO nuclear localization and decreases wing cell number, which recovers upon concurrent knockdown of *dfoxo* (A) Representative images of fat body dFOXO localization in *w*^*1118*^ and *ppl-Gal4>UAS-wap*^*i*^*Drosophila* (scale: 15μm). Fat body tissue was collected from wandering L3 larvae. (B) Representative images of adult wings (scale: 250 μm), with insets displaying wing hair density in a 10,000μm^2^ area. The outline of respective control wings is illustrated in magenta. (C–E) Quantification of wing area, wing cell number and wing cell area was performed using ImageJ and FijiWings software (n = 6–35 flies/group; mean ± SEM; *ns*: not significant). p value was calculated using two-way ANOVA with Tukey’s post-hoc. ∗∗p < 0.01, ∗∗∗∗p < 0.0001.

Given the importance of DCAF7 in maintaining both FOXO1 localization and cell proliferation, described above, we next explored whether DCAF7 regulation of FOXO1 is important for whole-organism growth. We used a *D. melanogaster* RNAi model system that is commonly employed to examine growth phenotypes. Importantly, DCAF7 and FOXO1 are conserved between species and share a high degree of sequence similarity when compared to their *Drosophila* orthologs Wap (wings apart, also known as CG14614 and Riquiqui) and dFOXO, respectively. dFOXO localization is frequently examined in the fat body of *Drosophila* L3 larvae, which is comparable to mammalian adipose tissue and liver. We found that RNAi knockdown of *wap* in the fat body, in *ppl-Gal4>UAS-wap*^*i*^ L3 larvae, prompted dFOXO nuclear localization in fat body cells (Figure 4A).

*Drosophila* wing area was used as an index of growth. Mutations in or depletion of *wap* in *Drosophila* result in growth retardation of the adult wing (Morriss et al., 2013; Yang et al., 2016), similar to that observed in flies overexpressing *dfoxo* (Kramer et al., 2003). Therefore, we examined whether the decrease in wing area induced by loss of *wap* was a result of enhanced dFOXO nuclear localization. RNAi knockdown of *dfoxo* in the wing, in *ap-Gal4>UAS-dfoxo*^*i*^ flies, did not alter wing area (Figure 4B and 4C), consistent with prior findings (Jünger et al., 2003). In contrast, *ap-Gal4>UAS-wap*^*i*^ wing area was significantly smaller compared to that in *UAS-wap*^*i*^ flies. The decrease in *ap-Gal4>UAS-wap*^*i*^ wing area was associated with a lower wing cell number (Figure 4D), without changing wing cell area (Figure 4E). Although *ap-Gal4>UAS-dfoxo*^*i*^*, UAS-wap*^*i*^ flies (with *wap* and *dfoxo* double knockdown) presented diminished wing area, they exhibited a significant gain in wing cell number compared to *ap-Gal4>UAS-wap*^*i*^, resembling that of control flies (Figure 4B–4D). Accordingly, wing cell area tended to decrease in *ap-Gal4>UAS-dfoxo*^*i*^*, UAS-wap*^*i*^ flies, although not reaching statistical significance (p = 0.09 compared to *UAS-dfoxo*^*i*^*, UAS-wap*^*i*^), precluding the recovery of wing size while wing cell number was reestablished (Figure 4E). Therefore, the reduced wing cell number in *wap* knockdown flies is mediated by dFOXO. These observations mirror those in flies lacking *chico* and in *chico; dfoxo* double knockout flies (Jünger et al., 2003), as *chico* knockout tempers wing area and cell number, whereas double knockout with *dfoxo* recovers cell number but is unable to increase wing area. Hence, our findings reveal a role for DCAF7 as a regulator of FOXO1 nuclear localization and cell proliferation *in vivo*.

## Discussion

The findings described identify DCAF7 as a novel interactor of IRS1. They also demonstrate that DCAF7 is a regulator of cell proliferation and cell cycle, as well as FOXO1 nuclear localization and expression of FOXO1-target genes. DCAF7 is a scaffold protein conserved in all eukaryotic genomes, with its amino acid sequence 100% conserved in mammals and 84% conserved between human DCAF7 and *Drosophila* Wap (Miyata et al., 2014; Nissen et al., 2006). DCAF7 is a substrate-specific adaptor of the DDB1-Cul4 E3 ubiquitin ligase complex, promoting proteasomal degradation of targeted proteins (Lee and Zhou, 2007; Liu et al., 2021; Peng et al., 2016). DCAF7 also regulates interactor levels independent of the proteasome (Kawara et al., 2019; Yousefelahiyeh et al., 2018). We did not detect changes in IRS1 protein abundance following DCAF7 knockdown, suggesting a functional role for the DCAF7-IRS1 interaction rather than mediating protein stability. This tenet is supported by previous work describing DCAF7 as an essential binding partner in various protein complexes, including with DYRK1A (Kawara et al., 2019; Miyata et al., 2014; Miyata and Nishida, 2011; Morita et al., 2006; Ritterhoff et al., 2010; Wang et al., 2018). In our experiments, the function of DCAF7 as an IRS1 scaffolding protein is supported by our finding that knockdown of DCAF7 decreases the interaction of IRS1 with DYRK1A. Moreover, coimmunoprecipitation identified that DCAF7 interacts with the p85 subunit of PI3K (Figure S4A). As such, DCAF7 may act in a similar manner to pleckstrin homology domain-interacting protein (PHIP), also known as DDB1- and CUL4-associated factor 14 (DCAF14), which has been shown by some groups to regulate IRS1 complex formation and insulin action (Farhang-Fallah et al., 2000, 2002). In addition, following DCAF7 knockdown, we find that phosphorylation of S612 in IRS1 increases (Figure S4B). S612 is a known inhibitory site that may explain the observed decrease in insulin signaling (Copps and White, 2012). Given that phosphorylation of ERK is unaffected by knockdown of DCAF7, it is unlikely that DCAF7 regulates the insulin receptor, rather, DCAF7 selectively regulates the IRS1-PI3K-AKT signaling arm. Thus, we speculate that DCAF7 operates as a scaffold in the formation of the IRS1-PI3K complex. However, future work is required to fully elucidate the mechanisms through which DCAF7 regulates signaling downstream of IRS1.

In mammalian cell lines, fish and flies, DCAF7 is implicated as a gene essential for cell survival (Alvarado et al., 2016; Hart et al., 2015; Miyata and Nishida, 2011; Morriss et al., 2013; Nissen et al., 2006; Ritterhoff et al., 2010; Yang et al., 2016; Yousefelahiyeh et al., 2018). Loss of DCAF7 lowers cell number over time, although this was suggested to be a result of more cell death rather than reduced cell division (Hart et al., 2015; Miyata and Nishida, 2011; Ritterhoff et al., 2010). In our hands, cell viability (LDH release) was eventually compromised at days 4 and 5 post transfection with DCAF7 siRNA (Figure S5A). Cell death was further assessed by flow cytometry with Annexin V and propidium iodide (PI). DCAF7 knockdown enhanced cell death akin to treatment with Staurosporine (Figures S5B and S5C). FOXO1 promotes transcription of apoptotic genes, including Bim (BCL2L11). Although FOXO1 nuclear localization is increased following DCAF7 knockdown, both *in vitro* and in *Drosophila*, we did not observe an increase in BCL2L11 expression (Figure S5D). Instead, our findings suggest that cell cycle arrest at G2 triggers the reduction in cell number following DCAF7 knockdown, and this is thereafter compounded by increased apoptosis. How DCAF7 knockdown promotes cell death has yet to be explored. One possibility is that cell cycle arrest may lead to cell death (Pucci et al., 2000).

Consistent with the above, FOXO1 activation is known to reduce cell proliferation (Accili and Arden, 2004; Greer and Brunet, 2005). Among FOXO1-target genes, GADD45A, CCNG2, CDKN1B, and RBL2 are genes that promote cell cycle arrest (Greer and Brunet, 2005). The elevated expression of the G2 cell cycle inhibitory genes GADD45A and CCNG2 upon DCAF7 knockdown correlates with G2 cell cycle arrest. FOXO1 is known to exhibit stimulus-dependent changes in gene expression, inducing upregulation of select target genes (Brunet et al., 2004; Huang and Tindall, 2007; Kobayashi et al., 2005; Motta et al., 2004). Thus, the selective increases in FOXO1 target genes observed here suggest that FOXO1 is not simply activated by DCAF7 knockdown, but also fine-tuned in a target-gene-specific manner. Given that GADD45A and CCNG2 exhibited enhanced expression, we propose that DCAF7 regulates cell proliferation via interaction with IRS1 and downstream growth factor signaling to FOXO1, culminating in transcriptional regulation of cell cycle inhibitors. In support of the DCAF7-IRS1 interaction and proximal signaling being responsible for observed FOXO1 regulation, insulin-stimulated AKT phosphorylation was attenuated by DCAF7 knockdown. Thus, DCAF7 regulation of FOXO1, via upstream interactions, points to DCAF7 as a mediator of the cellular transcriptional profile.

DYRK1A is a kinase and well-described interactor of DCAF7 (Miyata and Nishida, 2011; Yousefelahiyeh et al., 2018). Recent publications have identified DYRK1A as a regulator of both IRS1 (Tian et al., 2019) and FOXO1 (Bhansali et al., 2021; Woods et al., 2001). In neuronal cell lines and HEK293T cells, DYRK1A phosphorylates IRS1 and attenuates IRS1 degradation, leading to increased IRS1 protein content. Inhibition of DYRK1A thus resulted in decreased IRS1 protein (Tian et al., 2019). However, we find that knockdown of DCAF7 reduces DYRK1A content and the interaction of IRS1 with DYRK1A, while IRS1 protein levels remain unaltered. Moreover, in HepG2 cells, knockdown of DYRK1A does not attenuate insulin-stimulated phosphorylation of AKT S473 (Figures S6A–S6C) and therefore does not phenocopy DCAF7 knockdown. Thus, our findings suggest that DCAF7 regulates IRS1 independently of DYRK1A. Regarding FOXO1 regulation, DYRK1A binds FOXO1 in the nucleus and phosphorylates it on S329, an inhibitory phosphorylation site, in an insulin-independent manner (Bhansali et al., 2021; Woods et al., 2001). Thus, it is possible that DCAF7 knockdown could reduce DYRK1A inhibitory phosphorylation of FOXO1 and thus reduce cell proliferation. Yet DYRK1A knockdown induces a mild effect on cell number over time compared to DCAF7 knockdown (Figure 2A and S6D). Moreover, coimmunoprecipitation experiments show that FOXO1 and DCAF7 do not interact (Figure S4A); thus, DCAF7 is unlikely to act as a scaffold for DYRK1A regulation of FOXO1. Future work is required to examine whether reduced DYRK1A-mediated phosphorylation of FOXO1 on S329 contributes to increased FOXO1 activity and cell cycle arrest following DCAF7 knockdown.

DCAF7 is vital for the development of many model organisms (Degoutin et al., 2013; Morriss et al., 2013; Nissen et al., 2006; Ritterhoff et al., 2010) and is highly expressed during embryonic maturation (Bonano et al., 2018; Morita et al., 2006). DCAF7 is required for zebrafish craniofacial development (Nissen et al., 2006) and a genome-wide association study of single nucleotide polymorphisms in a multi-ethnic group of patients identified DCAF7 as a risk locus for cleft lip and cleft palate (Leslie et al., 2016). In *Drosophila*, DCAF7 is necessary for the development of various tissues (Degoutin et al., 2013; Morriss et al., 2013; Yang et al., 2016). The *Drosophila* and human genomes are 60% homologous, but notably the insulin signaling pathway as well as DCAF7 are highly conserved (Brogiolo et al., 2001; Mirzoyan et al., 2019; Nissen et al., 2006). Insulin signaling is a key determinant of body size and wing growth in *Drosophila* (Jünger et al., 2003; Kramer et al., 2003; Oldham et al., 2000). We show that *wap* knockdown elevated dFOXO nuclear localization in the larval fat body, and confirm that *wap* knockdown diminishes wing size (Degoutin et al., 2013; Yang et al., 2016). Furthermore, reduced wing size following *wap* knockdown is a result of decreased wing cell number, and this drop in cell number recovers upon parallel knockdown of *dfoxo*. Our findings mirror observations that the fall in wing cell number induced by *chico* knockout is suppressed by concurrent knockdown of *dfoxo*, while wing size remained small (Jünger et al., 2003). Overall, DCAF7 impacts on development, acting as a mediator of growth factor signaling.

In summary, we identify a previously unknown interaction between IRS1 and DCAF7, two scaffolding proteins implicated in cell survival. We further demonstrate that DCAF7 is, in turn, required for FOXO1 regulation. Loss of DCAF7 induces FOXO1 nuclear localization and promotes expression of G2 cell cycle inhibitor genes GADD45A and CCNG2, which are both FOXO1-targets. This correlates with the induction of G2 cell cycle arrest. In *Drosophila,* double knockdown of *wap* and *dfoxo* reversed the reduction in wing cell number observed following *wap* knockdown alone. We propose that DCAF7 is a mediator of IRS1 signaling important for cell proliferation and whole-organism growth. This finding adds insight into the fine-tuning of growth regulation through FOXO1.

### Limitations of the study

Given that we show DCAF7 regulates insulin signaling *in vitro*, and dFoxo localization in *Drosophila* tissue, a limitation of this study is that a role for DCAF7 in glucose metabolism and the development of insulin resistance was not examined. As IRS proteins are indispensable for insulin-stimulated glucose uptake, and both IRS and FOXO1 mediate insulin suppression of hepatic glucose production, it is interesting to speculate whether DCAF7 may contribute to insulin regulation of glucose homeostasis. Future studies are required to examine the impact of DCAF7 in glucose metabolism, insulin resistance, and type 2 diabetes.

## STAR★Methods

### Key resources table

**Table:** 

REAGENT or RESOURCE	SOURCE	IDENTIFIER
**Antibodies**		

Rabbit IRS1	Cell Signaling	Cat#2382
Rabbit pAKT S473	Cell Signaling	Cat#4060
Rabbit pAKT T308	Cell Signaling	Cat#9275
Mouse pan-AKT	Cell Signaling	Cat#2920
Rabbit FOXO1	Cell Signaling	Cat#2880
Rabbit DYRK1A	Cell Signaling	Cat#8765
Rabbit DCAF7	Sigma	Cat#HPA022948
Mouse Flag	Sigma	Cat#F3165
Mouse myc (9E10)	Santa Cruz	Cat#SC-40
Mouse GAPDH	Millipore	Cat#MAB374
Rabbit GFP	Thermo Fisher	Cat#A11122
Mouse pS10 Histone H3-488	Biolegend	Cat#650804

**Chemicals, peptides, and recombinant proteins**		

Dinaciclib	Selleckchem	Cat#S2768
Propidium iodine (PI)	Sigma-Aldrich	Cat#P4170
Lipofectamine RNAiMax	Invitrogen	Cat#13778150
Humulin R	Eli Lilly	Cat#U-100
GeneJuice	Millipore	Cat#70967
X-tremeGENE 9	Sigma-Aldrich	Cat#6365787001
Myc-Trap A	Chromotek	Cat#yta-20
EZview™ Red ANTI-Flag M2	Sigma-Aldrich	Cat#F2426
Tetracycline hydrochloride	Sigma-Aldrich	Cat#T7660
Biotin	BioShop	Cat#BIO302
Streptavidin Agarose	Genscript	Cat#L0035
Protease inhibitor cocktail	Sigma-Aldrich	Cat#P3840
Odyssey blocking buffer	LI-COR	Cat#927-50000
Annexin V-488	Thermo Fisher	Cat#A13201
Staurosporine	Sigma-Aldrich	Cat#S5921

**Critical commercial assays**		

Cytotoxicity Detection Kit (LDH)	Roche	Cat#11644793001

**Deposited data**		

IRS1 BioID MassIVE public repository (https://massive.ucsd.edu/)	This paper	MSV000088166 Username: MSV000088166_reviewer. Password: IRS1_BioID

**Experimental models: Cell lines**		

Human: HEK293T	ATCC	Cat#CRL-1573
Human: HepG2	ATCC	Cat#HB-8065
Human: HEK293-T-REx flip-in	Thermo Fisher	Cat#R78007

**Experimental models: Organisms/strains**		

*D. melanogaster:* RNAi of *dfoxo*: *y*^*1*^*v*^*1*^*; P{y*^*+*^*, v*^*+*^*, TRiP.JF02019}attP2*	Bloomington Drosophila Stock Center	BDSC: BL-25997
*D. melanogaster:* RNAi of *wings apart* (*wap*): *P{attP, y*^*+*^*, w*^*3*^*, KK102794}VIE-260B*	Vienna Drosophila RNAi Center	VDRC: v107076
*D. melanogaster: ppl-Gal4* driver: *w∗; P{w*^*+*^*, ppl-GAL4.P}2*	Bloomington Drosophila Stock Center	BDSC: BL-58768
*D. melanogaster: ap-Gal4* driver: *y*^*1*^*w*^*1118*^*; P{w*^*+*^*, GawB}ap*^*md544*^*/CyO*	Bloomington Drosophila Stock Center	BDSC: BL-3041

**Oligonucleotides**		

siRNA for DCAF7 (1)	Sigma Aldrich	SASI_Hs01_00066317
siRNA for DCAF7 (2)	Sigma Aldrich	SASI_Hs01_00235016
MISSION® siRNA Universal Negative Control (siNC)	Sigma Aldrich	#SIC001
Taqman CCNG2	Thermo Fisher	Hs00171119_m1
Taqman GADD45A	Thermo Fisher	Hs00169255_m1
Taqman RBL2	Thermo Fisher	Hs00180562_m1
Taqman CDKN1B	Thermo Fisher	Hs00153277_m1
Taqman Abt1	Thermo Fisher	Hs00706003_s1

**Recombinant DNA**		

IRS1-BirA∗Flag	This paper	N/A
IRS1myc	Ozoe et al. (2014)	N/A
Flag-hDCAF7	Glenewinkel et al. (2016)	N/A
GFP-rDYRK1A,	Glenewinkel et al. (2016)	N/A
GFP-hDYRK1B	Glenewinkel et al. (2016)	N/A

**Software and algorithms**		

Volocity	Perkin Elmer	N/A
Image Studio Version 3.1.4	LI-COR	N/A
SAINT Express v.3.3	SourceForge	N/A
X!Tandem	Craig and Beavis (2004)	N/A
ProteoWizard	Kessner et al. (2008)	N/A
MiKrowin 2000	Mikrotek Laborsystem GmbH	N/A
Fiji/ImageJ	Schneider et al., 2012	N/A
FijiWings	Dobens and Dobens (2013)	N/A
Prism	GraphPad	N/A

### Resource availability

#### Lead contact

Further information and requests for resources and reagents should be directed to and will be fulfilled by the lead contact, John Brumell (john.brumell@sickkids.ca).

#### Materials availability

All unique/stable reagents generated in this study are available from the lead contact with a completed Materials Transfer Agreement.

### Experimental model and subject details

#### Cell lines

Human embryonic kidney cells (HEK293T) were cultured in DMEM supplemented with 10% heat-inactivated FBS at 37°C and 5% CO_2_. The human hepatoma HepG2 hepatocyte cell line, derived from a male donor, was cultured in Low Glucose (5mM) DMEM supplemented with 10% FBS, which tonically activates IRS1.

#### Fly genetics

*D. melanogaster* were cultured on standard cornmeal molasses agar (Ashburner, 1989) at 25°C. RNAi stocks expressing hairpin RNAs directed against *dfoxo* (BL-25997: *y*^*1*^ *v*^*1*^*; P{y*^*+*^*, v*^*+*^*, TRiP.JF02019}attP2*) and *wings apart* (*wap*; v107076: *P{attP, y*^*+*^*, w*^*3*^*, KK102794}VIE-260B*) were acquired from Bloomington Drosophila Stock Center (BDSC) and Vienna Drosophila RNAi Center (VDRC), respectively. *dfoxo-wap* RNAi flies were generated using standard techniques. RNAi lines were expressed in fat body cells under control of the *ppl-Gal4* driver (BL-58768: *w∗; P{w*^*+*^*, ppl-GAL4.P}2*, BDSC) and in the wing disc under control of the *ap-Gal4* driver (BL-3041: *y*^*1*^ *w*^*1118*^*; P{w*^*+*^*, GawB}ap*^*md544*^*/CyO*, BDSC). Reporting of the sex and developmental stage of the flies used in each experiment is provided within the Method details section below.

### Method details

#### BioID sample preparation

BioID (Roux et al., 2012) was performed as described previously (Coyaud et al., 2015). In brief, full length human IRS1 coding sequence was amplified by PCR and cloned into pcDNA5 FRT/TO BirA∗Flag expression vector. Using the Flp-In system (Invitrogen), Flp-In 293 T-REx cells (Thermo Fisher Scientific) stably expressing FlagBirA∗ alone or IRS1-BirA∗Flag were generated. After selection (DMEM +10% FBS +200 μg/mL hygromycin B), two independent replicates of five 150 cm^2^ plates of sub-confluent (60%) cells were incubated for 24 h in complete medium supplemented with 1 μg/mL tetracycline (Sigma) and 50 μM biotin (BioShop). Cells were collected and pelleted (2,000 rpm, 3 min), the pellet was washed twice with PBS, and dried pellets were snap frozen. The cell pellet was resuspended in 10 mL of lysis buffer (50 mM Tris-HCl pH 7.5, 150 mM NaCl, 1 mM EDTA, 1 mM EGTA, 1% Triton X-100, 0.1% SDS, 1:500 protease inhibitor cocktail (Sigma-Aldrich), 1:1,000 benzonase nuclease (Novagen)) and incubated on an end-over-end rotator at 4°C for 1 h, briefly sonicated to disrupt any visible aggregates, then centrifuged at 45,000 × g for 30 min at 4°C. Supernatant was transferred to a fresh 15 mL conical tube. 30 μL of packed, pre-equilibrated Streptavidin-sepharose beads (GE) were added and the mixture incubated for 3 h at 4°C with end-over-end rotation. Beads were pelleted by centrifugation at 2,000 rpm for 2 min and transferred with 1 mL of lysis buffer to a fresh Eppendorf tube. Beads were washed once with 1 mL lysis buffer and twice with 1 mL of 50 mM ammonium bicarbonate (pH = 8.3). Beads were transferred in ammonium bicarbonate to a fresh centrifuge tube and washed two more times with 1 mL ammonium bicarbonate buffer. Tryptic digestion was performed by incubating the beads with 1 μg MS-grade TPCK trypsin (Promega, Madison, WI) dissolved in 200 μL of 50 mM ammonium bicarbonate (pH 8.3) overnight at 37°C. The following morning, 0.5 μg MS-grade TPCK trypsin was added, and beads were incubated 2 additional hours at 37°C. Beads were pelleted by centrifugation at 2,000 × g for 2 min, and the supernatant was transferred to a fresh Eppendorf tube. Beads were washed twice with 150 μL of 50 mM ammonium bicarbonate, and these washes were pooled with the first eluate. The sample was lyophilized and resuspended in buffer A (0.1% formic acid). 1/5th of the sample was analyzed per MS run.

#### Mass spectrometry

To perform mass spectrometry of BioID samples, high-performance liquid chromatography was conducted using a 2 cm pre-column (Acclaim PepMap 50 mm x 100 um inner diameter (ID)), and 50 cm analytical column (Acclaim PepMap, 500 mm x 75 um diameter; C18; 2 um; 100 Å, Thermo Fisher Scientific, Waltham, MA), running a 120 min reversed-phase buffer gradient at 225 nL/min on a Proxeon EASY-nLC 1000 pump in-line with a Thermo Q-Exactive HF quadrupole-Orbitrap mass spectrometer. A parent ion scan was performed using a resolving power of 60,000, then up to the twenty most intense peaks were selected for MS/MS (minimum ion count of 1,000 for activation) using higher energy collision induced dissociation (HCD) fragmentation. Dynamic exclusion was activated such that MS/MS of the same *m*/*z* (within a range of 10 ppm; exclusion list size = 500) detected twice within 5 s were excluded from analysis for 15 s. For protein identification, Thermo.RAW files were converted to the.mzXML format using Proteowizard (Kessner et al., 2008), then searched using X!Tandem (Craig and Beavis, 2004) and COMET (Eng et al., 2013) against the Human RefSeq Version 45 database (containing 36,113 entries). Data were analyzed using the *trans*-proteomic pipeline (TPP) (Deutsch et al., 2010; Pedrioli, 2010) via the Pro-Hits software suite (v3.3) (Liu et al., 2010). Search parameters specified a parent ion mass tolerance of 10 ppm, and an MS/MS fragment ion tolerance of 0.4 Da, with up to 2 missed cleavages allowed for trypsin. Variable modifications of +16@M and W, +32@M and W, +42@N-terminus, and +1@N and Q were allowed. Proteins identified with an iProphet cut-off of 0.9 (corresponding to ≤1% FDR) and at least two unique peptides were analyzed with SAINT Express v.3.6. Twenty control runs (from cells expressing the FlagBirA∗ epitope tag) were collapsed to the two highest spectral counts for each prey and compared to the two biological replicates (each with two technical replicates) of IRS1 BioID. High confidence interactors were defined as those with Bayesian false discovery rate (BFDR) ≤0.01.

#### Coimmunoprecipitation

HEK293T or HepG2 cells were seeded in 10 cm tissue culture dishes. The following day, cells were transfected with plasmids for 24 h using GeneJuice (#70967; Millipore) for HEK293T or X-tremeGENE 9 (#6365787001; Sigma-Aldrich) for HepG2 according to the manufacturers’ instructions. The following constructs were obtained as gifts: IRS1myc (from Dr. S.I. Takahashi, University of Tokyo, Japan; (Ozoe et al., 2014)), and GFP-rDYRK1A, GFP-hDYRK1B and Flag-hDCAF7 (from Dr. Walter Becker, Medical Faculty of RWTH Aachen, Germany; (Glenewinkel et al., 2016)). Co-immunoprecipitation was performed according to the manufacturer’s protocol for Myc-Trap_A (Chromotek) or EZview Red ANTI-Flag M2 (Sigma). Briefly, cells were washed twice with cold PBS and lysed in ice-cold lysis buffer (50 mM Tris-HCl, pH 7.4, 150 mM NaCl, 1 mM EDTA, 1% Triton X-100) supplemented with 1 mM PMSF, 5 mM NaF, 5 mM NaVO_4_, 10 μg/mL aprotinin 10 mg/mL, 10 μg/mL leupeptin, and 1 μM pepstatin A (Sigma). Lysates were centrifuged at 200,00× g for 10 min at 4°C and the supernatant was collected. Following protein quantification by BCA assay, 300 mg of protein was incubated with Myc-Trap_A (Chromotek) or EZview Red ANTI-Flag M2 (Sigma) for 2 h at 4°C with end-over-end tumbling. Beads were centrifuged at 2,500× g for 2 min at 4°C, washed 3× in Lysis Buffer, eluted with 50 μL of 2× SDS-sample buffer and boiled for 5 min prior to immunoblotting.

#### Immunoblotting

Immunoblotting was performed as previously described (Frendo-Cumbo et al., 2019). Briefly, cells were rinsed with cold PBS and lysed on ice in RIPA buffer (50 mM Tris, 150 mM NaCl, 1% Nonidet P-40, 0.5% Sodium Deoxycholate, 0.1% SDS) supplemented with 5 mM NaF, 1 mM EDTA, 1 mM phenylmethylsulfonyl fluoride (PMSF), 5mM Na3VO4 and protease inhibitors (#P3840; Sigma-Aldrich). Lysates were boiled for 5 min in 5× Laemmli sample buffer (LSB) with β-mercaptoethanol. Equal amounts of protein were separated by SDS-PAGE, transferred to nitrocellulose membranes, blocked with LI-COR Odyssey blocking buffer (#927–50000) and incubated overnight with primary antibodies in 1% BSA-TBST at 4°C with gentle agitation. The following primary antibodies were used: IRS1 (#2382), pAKT S473 (#4060), pAKT T308 (#9275), pan-AKT (#2920), pERK T202/Y204 (#9101), pP70S6K T389 (#9205), FOXO1 (#2880), β-actin (#4970), and DYRK1A (#8765) from Cell Signaling; DCAF7 (#HPA022948) and Flag (#F3165) from Sigma; anti-myc 9E10 (#SC-40) from Santa Cruz; GAPDH (#MAB374) from Millipore; GFP (#A11122) from Thermo Fisher. Membranes were then incubated with fluorescent secondary antibodies (IRDye 800CW or IRDye 680LT conjugated secondary; LI-COR) in TBST and developed using Odyssey Fc Imager (LI-COR). Results were quantified using the Image Studio 4.0 software.

#### siRNA transfection

For siRNA-mediated knockdown, HepG2 cells were transfected using Lipofectamine RNAiMax (#13778150; Invitrogen) as recommended by the manufacturer for 24 h siRNA was removed, and cells were grown for an additional 24 h in regular media containing serum, unless otherwise stated in the individual experiments. The following siRNAs were obtained from Sigma Aldrich: DCAF7(1) (SASI_Hs01_00066317), DCAF7(2) (SASI_Hs01_00235016) and siDYRK1A (SASI_Hs01_00123259). For control knockdown, MISSION siRNA Universal Negative Control (Sigma Aldrich, #SIC001) was used. When stated, serum deprivation was performed for 3 h, while insulin treatment (10 nM Humulin R U-100; Eli Lilly) was carried out for 10 min.

#### Cell cycle analysis

HepG2 cells growing in medium with serum were seeded in 6 well plates, treated for 24 h with siRNA and 72 h later trypsinized and spun at 400xg for 5 min. Dinaciclib (#S2768; Selleckchem) was used as a positive control for cell cycle inhibition (25 nM for 24 h). After two washes in PBS, cells were resuspended in 50 μL HBSS and transferred to a conical polypropylene tube containing 1 mL ice-cold 80% ethanol for 60 min at 4°C. Fixed cells were centrifuged at 400xg for 10 min and washed twice with PBS before resuspension in 1 mL of PBS containing 0.25% Triton X-100, for 15 min on ice. After a PBS wash, cells were incubated in anti-pS10 Histone H3-488 antibody (#650804; Biolegend) diluted in 1% BSA PBS for 90 min at room temperature. Cells were washed in PBS, resuspended in RNase A for 5 min and then supplemented with 0.1 mg/mL propidium iodine (PI) (#P4170; Sigma-Aldrich) solution for 30 min at room temperature. Cells were then filtered through Nitex into FACS tubes and immediately analyzed using Fortessa.

#### FOXO1 immunofluorescence on mammalian cells

For HepG2 siRNA knockdown experiments, cells were seeded in 6 well plates for 24 h prior to siRNA transfection with RNAiMAX for 24 h. Cells were then reseeded in 24-well plates with coverslips for an additional 24 h. Cells were serum deprived for 3 h, fixed and immunostaining was conducted as previously described (Frendo-Cumbo et al., 2019). Briefly, cells were permeabilized and blocked in PBS containing 0.2% saponin (Calbiochem) and 10% normal goat serum (SS-PBS) for 30 min. Subsequently cells were incubated for 1 h with FOXO1 antibody (#2880; Cell Signaling) in SS-PBS, washed three times with PBS and incubated with secondary Alexa Fluor conjugated antibodies and phalloidin-565 (Invitrogen) for 1 h. Cells were washed three times with PBS and mounted in fluorescence mounting medium (Dako). Images were acquired using a Quorum spinning disk confocal scan head (Leica DMI 6000 B inverted fluorescence microscope, Hamamatsu ORCA Flash 4 sCMOS and color camera) equipped with a 63× objective. FOXO1 nuclear localization was quantified using ImageJ software. Briefly, the nuclear compartment was marked using DAPI and the cell border was determined using phalloidin. The cytosolic region was isolated by removing the DAPI-nuclear mask from the phalloidin-whole cell mask. FOXO1 fluorescence was then measured in the nuclear and cytosolic regions and a ratio was calculated.

#### qPCR of FOXO1 target genes

RNA was purified using TRIzol reagent and cDNA was synthesized by reverse transcription using the Super-Script VILO cDNA kit (Thermo Fisher Scientific). cDNAs were amplified using predesigned TaqMan probes according to the manufacturer’s instructions. Taqman qPCR gene expression primers for cyclin G2 (CCNG2; Hs00171119_m1), growth arrest and DNA damage inducible alpha (GADD45A; Hs00169255_m1), RB transcriptional corepressor like 2 (RBL2; Hs00180562_m1), cyclin dependent kinase inhibitor 1B (CDKN1B; Hs00153277_m1), BCL2 like 11 (BCL2L11; Hs01076940_m1), DYRK1A (Hs00176369_m1) and activator of basal transcription 1 (Abt1; Hs00706003_s1) were from Thermo Fisher. Abt1 was used as a housekeeping gene and did not change between groups. Relative quantities of each mRNA were calculated by the comparative ΔΔCT method.

#### *Drosophila* fat body dissection and staining

Fat body was dissected from male and female wandering L3 larvae in cold PBS, pH 7.4. Dissected fat body was pooled (10–15 larvae/genotype) and fixed with 4% PFA PBS for 30 min at 4°C before permeabilization in PBST (PBS and 0.1% Triton X-100) at room temperature. The tissue was then blocked with 10% goat serum for 1 h at room temperature. Anti-dFOXO primary antibody (a gift from Dr. Pierre Leopold, Institut Curie, Paris, France; (Slaidina et al., 2009)) was diluted 1:200 in PBST with 10% goat serum, and fat body was incubated overnight at 4°C. Following three 10 min washes in PBST, fat body was incubated in Alexa Fluor 488 secondary antibody in PBST (1:500) for 1 h at room temperature. The fat body was then incubated in phalloidin 568 for 30 min at room temperature, followed by three 10 min washes in PBST, with DAPI diluted (1:1000) in the second wash. Samples were mounted in 10 μL Pro-Long Diamond (Thermo Fisher Scientific) using self-adhesive reinforcement labels (Avery #32203) as spacers. Mounted samples were cured overnight at room temperature before sealing with nail polish. Images were acquired using a Quorum spinning disk confocal scan head (Leica DMI 6000 B inverted fluorescence microscope, Hamamatsu ORCA Flash 4 sCMOS and color camera) equipped with a 63× objective.

#### Mounting of adult wings

Wing mounting was performed as previously described (Eivers et al., 2009), with slight variations. Briefly, wings were removed from adult female flies and placed directly on a glass slide with 50% ethanol for dehydration. After ethanol evaporation, 30μL of Canada Balsam was placed on the slide and a glass coverslip was placed on top. The slide was placed on a metal surface and a magnet placed on top for 10min to flatten the preparation. The slide was then transferred to an oven set to 65°C for 15min prior to being sealed with nail polish. Slides were imaged using 3DHistech slide scanner (Quorum Technologies). Wing area and wing cell number were measured using ImageJ and FijiWings software and wing cell area was quantified as previously described (Brogiolo et al., 2001; Dobens and Dobens, 2013; Jünger et al., 2003). Briefly, as each wing hair represents a single cell, wing hairs were counted in a 10,000 μm^2^ area just posterior to the posterior cross vein, in the distal wing intervein region between longitudinal veins L4 and L5. The cell density was then multiplied by the wing area to approximate the number of cells in the whole wing.

#### LDH assay

A Cytotoxicity Detection Kit (LDH) (Roche) was used to assess media lactate dehydrogenase (LDH) activity, as a measure of cell viability. On days 1–5 post siRNA transfection cell culture medium was collected and centrifuged at 15,000xg for 10 min. Supernatants were transferred to 96-well plates in a 1:1 ratio with the test solution and read on a microplate reader at an absorbance of 490 nm, using 690 nm as a background measurement. LDH activity was first made relative to total LDH activity within each siRNA treatment group, measured by triton treatment of cells to induce LDH release prior to collecting media. This was then expressed relative to the non-coding control siRNA group.

#### Cell death analysis

HepG2 cells were seeded in 6 well plates, treated for 24 h with siRNA and 96 h later media was collected, and cells were trypsinized, transferred to an Eppendorf tube containing collected media and spun at 400xg for 5 min at 4°C. Staurosporine (#S5921; Sigma-Aldrich) was used as a positive control to induce apoptosis (1 μM for 1 h). After two washes in cold PBS, cells were resuspended in 100μL Annexin binding buffer (10 mM HEPES pH 7.4, 140 mM NaCl and 2.5 mM CaCl2). Annexin V-488 (#A13201; Thermo Fisher) was added to the 100μL cell suspension for 15 min. After two washes in PBS, cells were resuspended in 100μL Annexin binding buffer supplemented with 1μL 100 μg/mL propidium iodine (PI). After 15 min, cells were diluted with 400μL Annexin binding buffer filtered through Nitex into FACS tubes and immediately analyzed using Fortessa.

### Quantification and statistical analysis

Statistical analysis was conducted using GraphPad Prism v.7.0g. The mean ± SEM is shown in figures and the statistical tests used to determine p values are described in detail in corresponding figure legends. p < 0.05 was considered statistically significant. The degree of significance is denoted as follows: ∗p < 0.05, ∗∗p < 0.01, ∗∗∗p < 0.001, ∗∗∗∗p < 0.0001.

## Data Availability

• Raw mass spectrometry data for BioID analysis of IRS1 has been uploaded to the MassIVE public repository (https://massive.ucsd.edu/) under accession# MSV000088166. • Proximity interactions for IRS1-BirA∗-Flag compared to Flag-BirA∗ BioID alone performed under similar conditions (1% FDR) are presented in Data S1. • This paper does not report original code. • Any additional information required to reanalyze the data reported in this paper is available from the lead contact upon request.

## Supplemental information

Document S1. Figures S1–S6

Data S1. Proximity interactions for IRS1-BirA∗-Flag compared to control data from Flag-BirA∗ performed under similar conditions
