# DCAF7 regulates cell proliferation through IRS1-FOXO1 signaling

**DOI:** 10.1016/j.isci.2022.105668

**Published:** 2022-12-05

**Authors:** Scott Frendo-Cumbo, Taoyingnan Li, Dustin A. Ammendolia, Etienne Coyaud, Estelle M.N. Laurent, Yuan Liu, Philip J. Bilan, Gordon Polevoy, Brian Raught, Julie A. Brill, Amira Klip, John H. Brumell

## Main text

(iScience *25*, 105188; October 21, 2022)

On examining the online pdf, the authors discovered a mistake in two of the labels in the graphical abstract. The labels "*UAS-wap*" and "*ap-GAL4>UAS-wap*" should both have a superscript "*i*" after "*wap*" (i.e., *wap* should be replaced with *wap*^*i*^) to indicate that the transgene induces RNAi rather than expression of the *wap* gene. This has now been corrected. The authors regret this error and apologize for any confusion that it has caused.

