# Supplementary material for: DCAF7 regulates cell proliferation through IRS1-FOXO1 signaling
Source: iScience. 2022 Sep 24;25(10):105188. doi: 10.1016/j.isci.2022.105188 (PMC9556925; doi:10.1016/j.isci.2022.105188)
Supplement: Document S1. Figures S1–S6 [file mmc1.pdf]

iScience, Volume *25*

Supplemental information

DCAF7 regulates cell proliferation through IRS1-FOXO1 signaling

Scott Frendo-Cumbo, Taoyingnan Li, Dustin A. Ammendolia, Etienne Coyaud, Estelle M.N. Laurent, Yuan Liu, Philip J. Bilan, Gordon Polevoy, Brian Raught, Julie A. Brill, Amira Klip, and John H. Brumell

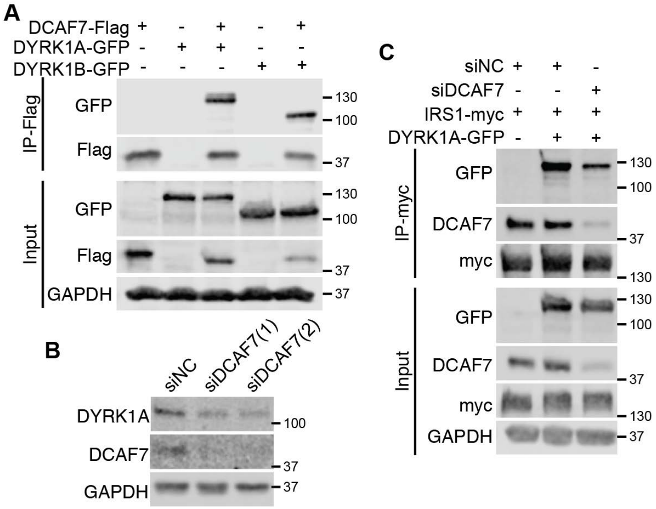

Figure S1. DCAF7 promotes the interaction of IRS1 with DYRK1A. Related to Figure 1.
(A) Coimmunoprecipitation of DYRK1A-GFP and DYRK1B-GFP with DCAF7-Flag in HepG2 cells.
(B) DYRK1A protein abundance measured by western blotting following transfection with non-coding (NC) or two independent DCAF7 siRNA.
(C) Coimmunoprecipitation of DYRK1A-GFP and endogenous DCAF7 with IRS1-myc in NC or DCAF7 siRNA transfected HEK293T cells.

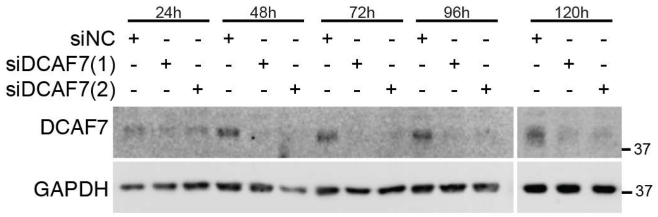

Figure S2. DCAF7 is efficiently knocked down 24h-120h post transfection with two independent siRNA sequences. For 120h post transfection with siRNA, the representative blot was run on a separate gel from other time-points. Related to Figure 2 and Figure 3.

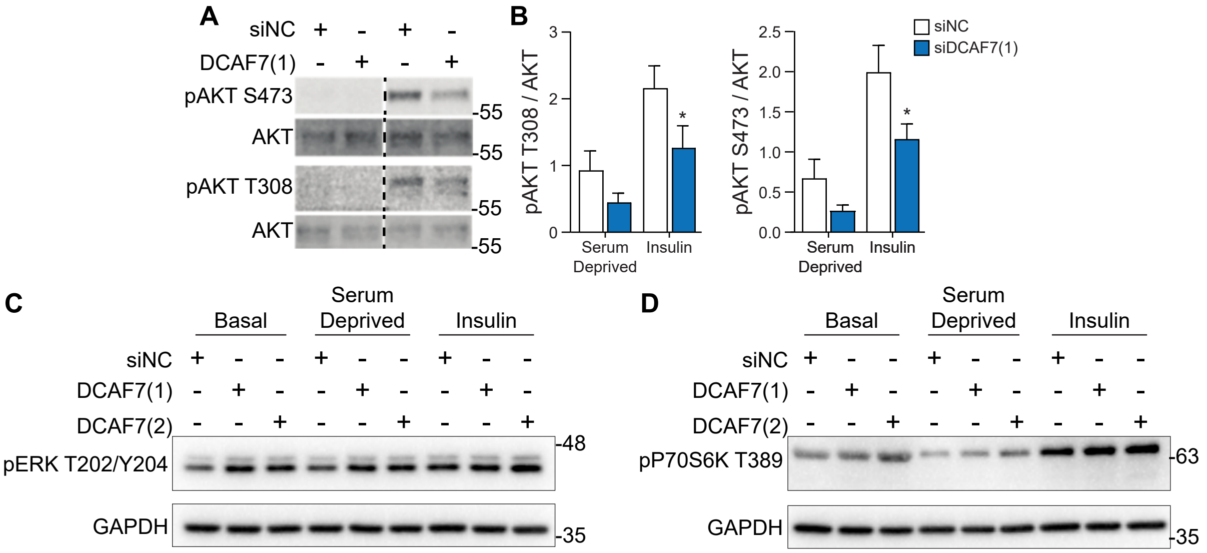

Figure S3. DCAF7 knockdown attenuates insulin stimulated phosphorylation of AKT, but not ERK or P70S6K. Related to Figure 3.

(A)-(B) Cells were serum deprived for 3 hours prior to treatment with insulin, and phosphorylation of AKT on S473 and T308 was examined by western blotting (n = 3/group; mean ± SEM). P value was calculated using a two-way ANOVA with Tukey's post-hoc. The representative blot is from the same membrane, cropped to show only relevant groups.

(C) Cells were maintained in regular media (Basal) or serum deprived for 3 hours prior to treatment with insulin, and phosphorylation of ERK on T202/Y204 was examined by western blotting.

(D) Cells were maintained in regular media (Basal) or serum deprived for 3 hours prior to treatment with insulin, and phosphorylation of P70S6K on T389 was examined by western blotting.

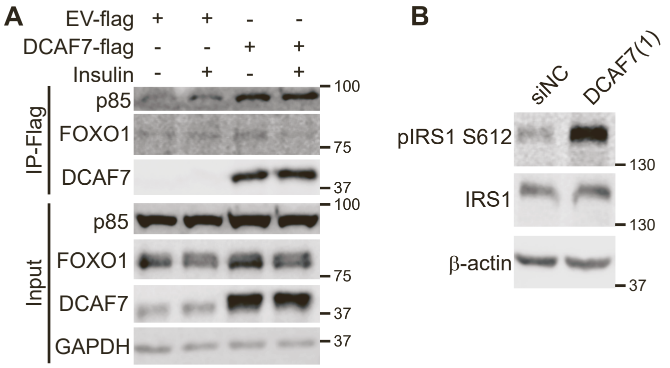

Figure S4. DCAF7 coimmunoprecipitates with p85, but not FOXO1, and loss of DCAF7 promotes inhibitory IRS1 phosphorylation on S612. Related to Figure 3.

(A) Coimmunoprecipitation of DCAF7-Flag displays an interaction with endogenous p85, but not FOXO1, in the presence or absence of insulin in HepG2 cells.

(B) HepG2 cells were transfected with non-coding (NC) or DCAF7 siRNA 48 hours prior to experimentation. Phosphorylation of IRS1 on S612 was examined by western blotting.

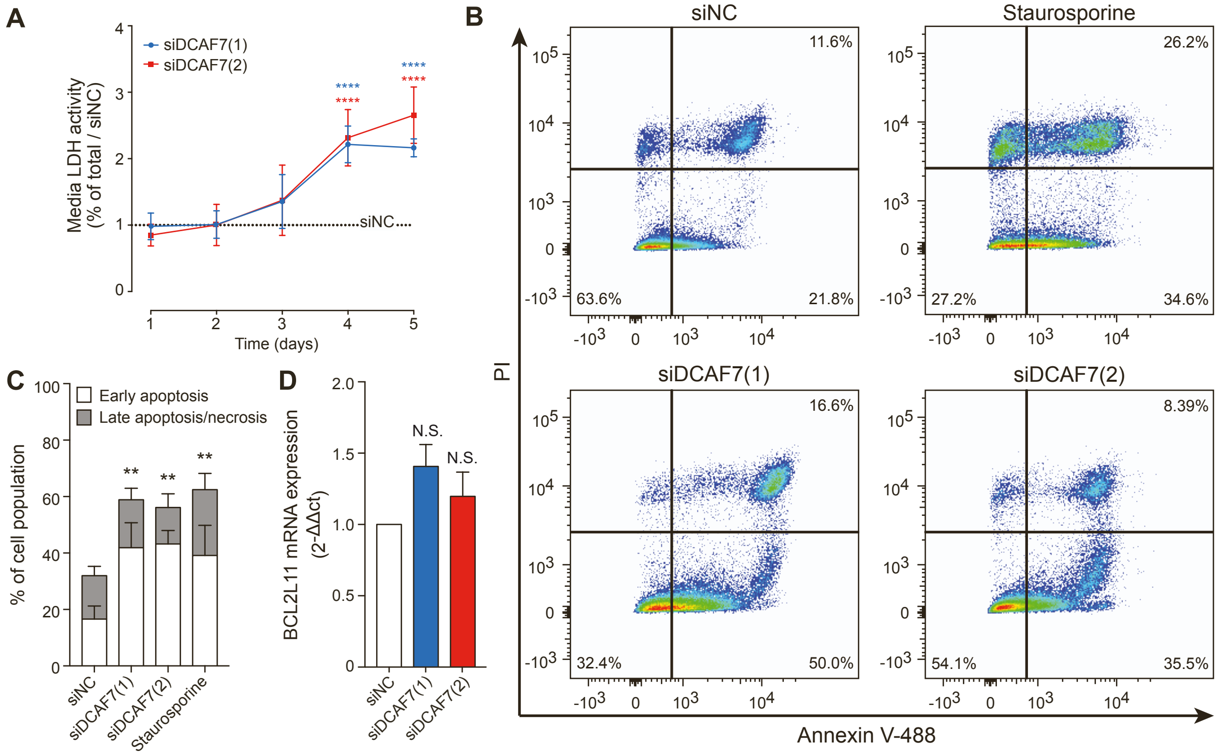

Figure S5. DCAF7 knockdown promotes apoptotic cell death. Related to Figure 2.
(A) Media LDH activity assay was performed to assess cellular viability in NC and DCAF7 siRNA treated cells (n = 3/group; mean ± SEM). P value was calculated using two-way ANOVA with Fisher's LSD post-hoc. See also Figure S2.
(B) Representative plots of cell death analysis following siRNA transfection. Transfected cells were stained with Annexin V-488 and PI prior to flow cytometry for analysis of cell death. Cells that are Annexin V positive and PI negative are considered to be in early apoptosis, while cells positive for both stains are considered to be in late apoptosis or necrosis. Cells treated with Staurosporine (1 µM for 1 hour), a pharmacological inducer of apoptosis, were used as a positive control.
(C) Quantification of the percent of cell population in early apoptosis and late apoptosis/necrosis (n = 3/group; mean ± SEM). P value was calculated using one-way ANOVA with Fisher's LSD post-hoc. $^{**}P < 0.01$, $^{****}P < 0.0001$.
(D) Expression of BCL2L11, a FOXO1-regulated apoptotic gene, was examined by RT-qPCR (n = 3/group; mean ± SEM).

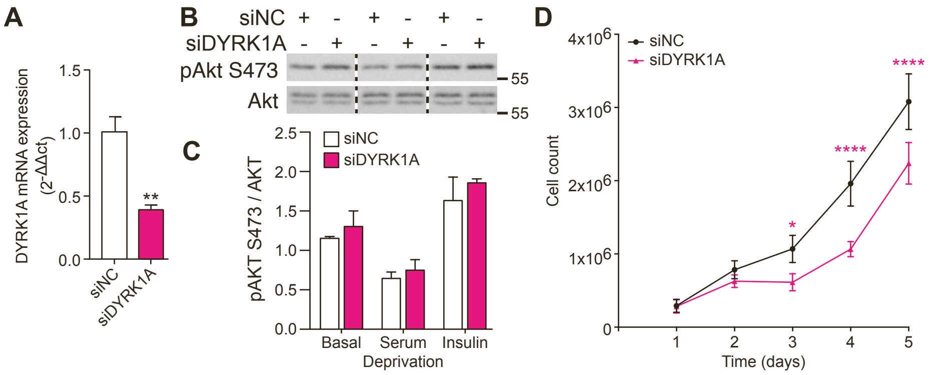

Figure S6. DYRK1A knockdown does not phenocopy the impact of DCAF7 knockdown on AKT phosphorylation or cell number. Related to Figure 1, Figure 2 and Figure 3.
(A) DYRK1A is efficiently knocked down 48h post transfection with siRNA.
(B)-(C) Cells were serum deprived for 3 hours prior to treatment with insulin, and phosphorylation of AKT on S473 and T308 was examined by western blotting (n = 3/group; mean ± SEM). P value was calculated using a two-way ANOVA with Tukey's post-hoc. The representative blot is from the same membrane, cropped to show only relevant groups.
(D) HepG2 cell number 1-5 days post transfection of NC or DYRK1A siRNA (n = 5/group; mean ± SEM). Cells were grown in the presence of serum. P value was calculated using two-way ANOVA with Fisher's LSD post-hoc. *P < 0.05, **P < 0.01, ****P < 0.0001.
